# Incorporating circulating cytokines into the idiopathic inflammatory myopathy subclassification toolkit

**DOI:** 10.3389/fmed.2023.1130614

**Published:** 2023-03-16

**Authors:** Boel De Paepe

**Affiliations:** Laboratory for Neuropathology, Department of Neurology and Neuromuscular Reference Center, Ghent University Hospital, Ghent, Belgium

**Keywords:** idiopathic inflammatory myopathy, circulating biomarker, creatine kinase, growth differentiation factor 15, C-X-C motif chemokine 10

## Abstract

Extensive diagnostic delays and deferred treatment impact the quality of life of patients suffering from an idiopathic inflammatory myopathy. In-depth subtyping of patients is a necessary effort to engage appropriate disease management and may require specialized and elaborate evaluation of the complex spectrum of clinical and pathological disease features. Blood samples are routinely taken for diagnostic purposes, with creatine kinase measurement and autoantibody typing representing standard diagnostic tools in the clinical setting. However, for many patients the diagnostic odyssey includes the invasive and time-consuming procedure of taking a muscle biopsy. It is proposed that further implementation of blood-based disease biomarkers represents a convenient alternative approach with the potential to reduce the need for diagnostic muscle biopsies substantially. Quantification of judicious combinations of circulating cytokines could be added to the diagnostic flowchart, and growth differentiation factor 15 and C-X-C motif chemokine ligand 10 come forward as particularly good candidates. These biomarkers can offer complementary information for diagnosis indicative of disease severity, therapeutic response and prognosis.

## 1. Introduction

Notwithstanding their classification as rare conditions, idiopathic inflammatory myopathies (IIM) represent a diverse group of diseases. Main subgroups include dermatomyositis (DM), polymyositis (PM), sporadic inclusion body myositis (IBM), immune-mediated necrotizing myopathy (IMNM), and myositis as part of the anti-synthetase syndrome (ASS) (1). For DM patients who exhibit the characteristic muscle weakness with cutaneous manifestations a diagnosis can swiftly be made, however definitive allocation to most IIM subgroups can be a challenge. Often, taking a muscle biopsy to look for disease-associated hallmark myopathological characteristics remains a diagnostic necessity. Well-thought-out sets of histological and immunohistochemical stainings can reveal the distinguishing patterns of muscle fiber damage and inflammation that differ between the IIM (2). Characteristic capillary damage and perifascicular muscle fiber atrophy may typically be observed in DM, along with inflammatory infiltrates composed of plasmacytoid dendritic cells, T-cells, macrophages and B-cells located predominantly at the perimysium. In biopsies from PM and IBM patients, inflammation typically builds up at endomysial sites, with nonnecrotic muscle fibers becoming invaded by auto-aggressive cytotoxic T-cells and macrophages. PM is regarded as an exceedingly rare diagnosis, as often disease characteristics are recognized, or seen to evolve toward other inflammatory myopathy subclassifications, including IBM (3). In IBM, muscle fibers additionally develop degenerative changes, represented by the formation of rimmed vacuoles and aggregated protein inclusions. In biopsies from IMNM patients on the other hand, muscle fiber necrosis predominates over inflammatory cell accumulation (4).

Subclassification of the IIM is by no means a redundant effort, as prognosis and therapeutic response differ substantially between groups. While the cornerstone of therapy for PM is immunosuppression with corticosteroids, IBM patients are usually resistant to immunomodulatory therapy. Complicating matters further, the clinical picture and pathological features of a hereditary muscle disorder may resemble an IIM. In patients diagnosed with a muscular dystrophy for instance, inflammation is often a prominent feature which interdependently with genetic mechanisms influences disease status profoundly. The need persists for improvements to diagnostic procedures, especially focused at reducing the diagnostic delay and preventing unnecessary exposure of patients to inappropriate hence ineffective and potentially harmful therapies. Innovative diagnostic procedures that reduce the need for taking a skeletal muscle biopsy, an invasive technique that lengthens the diagnostic process, would need to compensate for the loss of information which would have been provided by such a biopsy, including information on disease pathology and severity. The search for specific blood-based disease biomarkers for IIM is an ongoing process (5), and the conspicuous subtype-specific variability of IIM’s common denominator of chronic inflammation makes that detailed typing of circulating inflammatory factors represents an attractive strategy with potential added value.

## 2. Standard care blood biomarkers

Currently, when a patient is suspected of having an IIM, a relatively limited set of blood biomarkers is routine checked in clinical practice. Creatine kinase (CK) quantification is the most widely used blood marker both to diagnose and to follow-up muscle disease. CK is present in small amounts in the blood of a healthy individual, yet leaks into the bloodstream when muscle tissue gets damaged. Any condition that leads to muscle damage, or interferes with muscle energy production, which includes strenuous exercise, may therefore lead to increased CK levels. CK quantification cannot differentiate IIM patients from patients with other myopathies such as hereditary muscular dystrophies, who generally exhibit high blood CK levels (6), nor can it firmly differentiate between IIM subtypes. Nonetheless, some subgroups have a tendency toward higher (IMNM) and other to near-normal (IBM) CK levels. In addition to its diagnostic purpose, determining serum CK levels can be a useful marker for evaluating IIM disease activity. This should also be handled with caution, as CK levels have been described as unrelated to muscle weakness or disease severity in DM patients (7), and in IBM are most often normal or only slightly increased (8). In the majority of IIM patients, levels of transaminases strongly correlate with CK levels (9) and alanine aminotransferase (ALT) and aspartate aminotransferase (AST) are common blood biomarkers for IIM. These enzymes are present in the liver and in the skeletal muscle tissue, and circulate only in small amounts in healthy individuals. When the levels of both enzymes are increased, this is most indicative of liver injury. Elevated AST levels and normal ALT levels are more likely to be associated with conditions of muscle, heart, or kidney. Transaminases may be elevated in the early phase of muscle disease preceding characteristic symptoms that allow proper diagnosis. When serum ALT/AST is elevated, determining gamma-glutamyl transferase and CK are helpful to firmly differentiate liver from muscle disease (10). In addition to transaminases, lactate dehydrogenase (LDH) may be determined. Increased levels of the glycolytic pathway enzyme aldolase, found in all tissues but most predominantly in skeletal muscle, liver, and brain, can be helpful especially for identifying IIM patients with normal blood CK levels (11). Combining blood biomarkers represents true added value for diagnosis, and a combination of CK, AST and aldolase has been proposed as a convenient strategy (12).

## 3. Autoantibodies

In recent years, profiling of circulating autoantibodies in patients has gained importance for diagnosis of IIM subtypes. While myositis-associated antibodies, including anti-PM/Scl, anti-Ro52 and anti-U1RNP, may also be present in other conditions, myositis-specific autoantibodies (MSA) are especially revealing for IIM diagnosis. In fact, a distinct subgroup within the IIM has been defined in recent years based upon the very presence of anti-synthetase autoantibodies termed ASS. Patients with ASS display distinct clinical features that may include interstitial lung disease (ILD), Raynaud’s phenomenon, and arthritis (13). These patient carry one of eight anti-synthetase autoantibodies identified so far: anti-histidyl-tRNA synthetase (anti-Jo-1), anti-alanyl-tRNA synthetase (anti-PL-12), anti-threonyl-tRNA synthetase (anti-PL-7), anti-glycyl-tRNA synthetase (anti-EJ), anti-isoleucyl-tRNA synthetase (anti-OJ), anti-asparaginyl-tRNA synthetase (anti-KS), anti-phenylalanyl-tRNA synthetase (anti-Zo), and anti-tyrosyl-tRNA synthetase (anti-Ha/YRS) (14).

DM is associated with an intriguing diversity of MSA that often have characteristic associated systemic and cutaneous manifestations. Most common DM-associated MSA, which are detected in a range of 60 to 80% of patients, are directed against nuclear helicase (anti-Mi-2), type 5 protein associated with melanoma (anti-MDA-5), nuclear matrix protein 2 (anti-NXP-2), transcriptional intermediary factor 1 γ (anti-TIF-1γ), and small ubiquitin-like modifier activating enzyme (anti-SAE-1/2). Typing of these autoantibodies helps to predict disease phenotypes and offers valuable prognostic information. Anti-MDA5 and anti-Ro-52 autoantibodies for instance associate with DM/PM with ILD, while patients seropositive for anti-TIF1γ and anti-NXP2 are less likely to develop ILD. When anti-NXP2, anti-SAE, and most particularly anti-TIF1-γ autoantibodies are found, patients should be very closely monitored for DM-associated malignant comorbidities. In contrast, most studies report low prevalence of malignancy in patients with anti-Mi-2 autoantibodies (15).

In view of the potentially confounding symptoms, detection of autoantibodies can be very valuable to correct an initial diagnosis of limb girdle muscular dystrophy to IMNM (16). Moreover, subtyping *via* autoantibody profiling is of particularly value for IMNM patients, as the disorder’s variable disease phenotype, severity and treatment response have been linked with autoantibody status (4). The most prevalent autoantibody in IMNM is directed against 3-hydroxy-3-methylglutaryl-CoA reductase (HMGCR), an enzyme that catalyzes the conversion of HMG-CoA to the cholesterol precursor mevalonate. A subset of IMNM patients has autoantibodies against HMGCR, part of whom had used statins as a cholesterol-lowering drug. Anti-HMGCR positive IMNM is usually associated with moderate muscle weakness. Auto-antibodies directed against signal recognition particle (SRP) also strongly associate with IMNM, and anti-SRP positive patients usually display more severe and rapidly progressing weakness in proximal more than in distal muscles. Autoantibody-negative IMNM forms a subgroup of 20 to 30% of patients with distinctive clinical features (17), whom along with anti-HMGCR seropositive IMNM patients are at greater risk of developing malignancies than anti-SRP seropositive patients. In IMNM with anti-SRP (18) or anti-HMGCR (19) autoantibodies, CK levels correlate strongly with myofiber necrosis and disease activity and can be used in the follow-up of patients. In the active phase of IMNM, anti-HMGCR seropositivity often shows ALT predominance, whereas in anti-SRP positive patients AST often predominates (20).

An autoantibody associated with IBM has also been recognized, which is detected in up to 70% of patients and is directed against cytosolic 5′-nucleotidase 1A (NT5c1A) (21–23), and has quickly gained status as a diagnostic biomarker (24). Anti-NT5c1A has a moderate sensitivity, yet displays high specificity for IBM, although recent data showed presence of anti-NT5c1A also in other autoimmune conditions, particularly in systemic lupus erythematosus and Sjogren’s syndrome (25). Nonetheless, anti-NT5c1A represents a valuable supportive diagnostic criterion for IBM alongside clinical signs and pathological findings that could remain inconclusive.

## 4. Circulating cytokines as novel biomarkers

In chronic skeletal muscle tissue inflammation, inflammatory factors may reach the circulation, revealing the specific immunological characteristics of the underlying immune condition. Our knowledge of the specific cytokine profiles of IIM subgroups is ever expanding. Within diagnostic subgroups, it has been observed that patients with different MSA seropositivity had unique cytokine expression patterns that correlate with clinical indices in the blood (26). A recent review of myositis biomarkers incorporates a summary of established knowledge of cytokine expression as well as more novel findings, elegantly showing their growing diagnostic purpose (27). Quantifying cytokines, chemokines, and immune-related growth factors in patient sera represents a convenient diagnostic approach which can be achieved with an enzyme-linked immunosorbent assay, an accessible highly sensitive and reproducible technique for which many kits are commercially available. Our recent studies determined blood levels of a set of cytokines in IBM and IMNM patients (Table 1). At our neurology department, most patients that present with a diagnosis of inflammatory myopathy are determined to fit into these two subgroups, hence we focused research on pre-treatment IBM and IMNM blood samples. Our studies singled out growth differentiation factor 15 (GDF-15) and C-X-C motif chemokine ligand 10 (CXCL10) as biomarkers with possible added value for diagnosis (28, 29). GDF-15 is an injury-associated cytokine expressed and secreted in response to oxidative stress and inflammation, and CXCL10 is a chemokine, also known as Interferon gamma-induced protein 10 (IP-10), involved in the recruitment of macrophages, dendritic cells, natural killer cells and activated T lymphocytes toward areas of inflammation. Evidence progressively accumulates of circulating CXCL10 representing a valuable diagnostic biomarker for different subgroups of IIM (30–34), and increased levels of GDF-15 have been confirmed in IBM patients (35).

**Table 1:** Serum biomarker levels in patients.

	HC (*n* = 10)	IMNM (*n* = 10)	IBM (*n* = 10)	LGMDR (*n* = 2)	References
CK (u/l)	ND	2,807 ± 3,480	324 ± 225	3,206 ± 2,332	(28)
GDF-15 (pg/mL)	326 ± 204	555 ± 368	800 ± 288	106 ± 21	(29)
CXCL10 (pg/mL)	79 ± 53	360 ± 362	1,021 ± 681	110 ± 11	(28)
CCL5 (pg/mL)	2073 ± 1,510	42,358 ± 22,328	39,551 ± 27,693	27,173 ± 10,746	(28)
CD40L (pg/mL)	74 ± 207	6,926 ± 2,473	7,506 ± 2,351	9,068 ± 426	(28)

Mean ± SD serum levels of creatine kinase (CK), growth differentiation factor 15 (GDF-15), C-X-C motif chemokine 10 (CXCL10), C-C motif ligand 5 (CCL5) and cluster of differentiation 40 ligand (CD40L) in healthy controls (HC) and patients diagnosed with immune-mediated necrotizing myopathy (IMNM), sporadic inclusion body myositis (IBM) and limb girdle muscular dystrophy recessive inheritance patients diagnosed with LGMDR12 ANO5 and LGMDR25 POPDC1. Not determined (ND). All patients were treatment naïve and had active disease at the time of sampling.

## 5. Combining serum autoantibodies and CK with GDF-15 and CXCL10 levels

**Figure 1:**
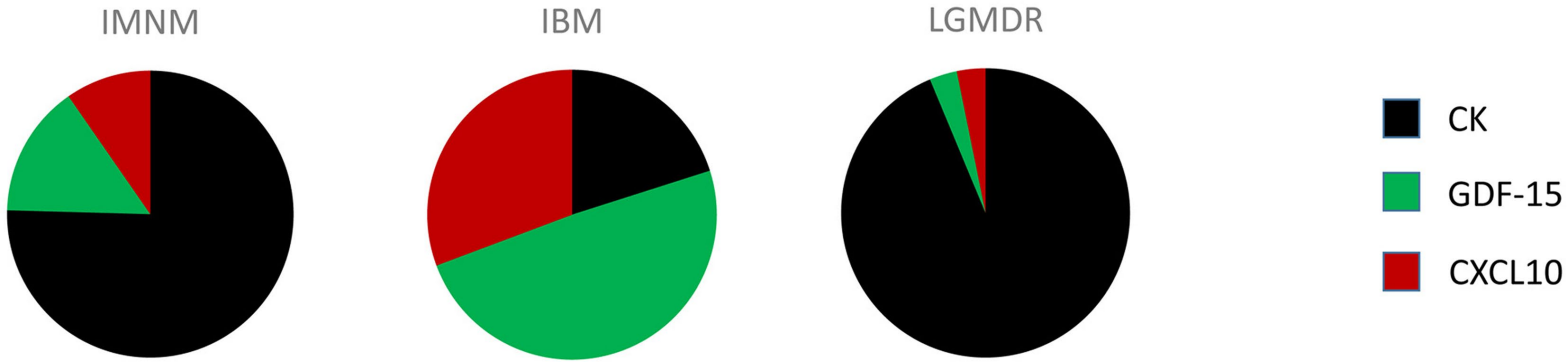
Comprehensive quantitative representation of the relative importance of creatine kinase (CK), growth differentiation factor 15 (GDF-15), and C-X-C motif chemokine 10 (CXCL10) serum levels in patients diagnosed with immune-mediated necrotizing myopathy (IMNM), sporadic inclusion body myositis (IBM) and limb girdle muscular dystrophy recessively inherited (LGMDR) patients diagnosed with LGMDR12 *ANO5* and LGMDR25 *POPDC1*.

Autoantibody profiling has firmly taken its place in the diagnostic workup of IIM, however in an average of 40% of IIM patients no MSA can be detected. It is highly unlikely that a single circulating biomarker can be identified which is capable of diagnosing and/or subtyping IIM, yet a clever combination of biomarkers could potentially do the trick. As has been stated by Reed et al. in the context of juvenile IIM, individual biomarkers may provide useful information yet a concerted effort may be necessary to augment their use for clinical practice (36). Combining autoantibody profiling with quantification of CK, GDF-15 and CXCL10 in patient blood samples is proposed as a diagnostic strategy for IIM which would be able to reduce the need for skeletal muscle tissue evaluation. Analysis of relative expression levels of these three disease-associated factors could help identify and subtype patients (Figure 1). From our own research, the following scheme can be proposed: low end CK and high end GDF-15 and CXCL10 levels point toward IBM, high end CK and intermediate GDF-15 and CXCL10 levels point toward IMNM, and high end CK and low end GDF-15 and CXCL10 point toward a muscular dystrophy. This strategy is based upon our current knowledge and would, of course, need to be confirmed in larger patient cohorts. In view of the rarity of these conditions, we were unable to collect sufficient samples from DM, ASS and PM, which could be remedied by international cooperation initiatives such as the International Myositis Assessment & Clinical Studies Group (IMACS) (37). In addition, other subgroups of IIM and hereditary muscle disorders with overlapping symptoms should be included. However, it can be proposed with confidence that combining quantification of cytokines and CK with autoantibody profiling in particular might represent a diagnostic strategy better able to differentiate IIM from other muscle disorders and to subtype patients to groups relevant to treatment and prognosis.

## 6. Discussion

Only in a minority of cases, a diagnosis of IIM can swiftly be made based upon characteristic clinical muscle and non-muscle signs. An example are DM patients with characteristic muscle complaints and the hallmark skin lesions. However, DM patients may also present with muscle weakness only (DM sine dermatitis) or solely exhibit cutaneous manifestations (amyopathic DM) (38). In IIM patients, diagnosis most often requires combining data of a patient’s clinical signs with serological, pathological and genetic analyses. As much as a skilled neuropathologist is essential for the diagnosis of IIM, such an expert may be a rare find. This results in increased diagnostic delays and deprives patients from the timely startup of therapies. The medical community awaits more convenient biomarkers able to reduce the necessity of taking diagnostic muscle biopsies. In DM, it has been shown that typing of autoantibodies yields information directly associated with specific muscle pathology characteristics. Anti-TIF1-γ seropositivity for instance associates with fiber vacuolation and perifascicular expression of Major Histocompatibility Complex class I, anti-Mi-2 with perifascicular muscle fiber damage and sarcolemmal membrane attack complex deposition, and anti-MDA5 with Myxovirus resistance A staining (39). For characterization into distinct IIM subgroups, typing of autoantibody profiles however also does not suffice, as a substantial group of patients are autoantibody negative.

There is also a need for diagnostic biomarkers that can reliably differentiate IIM from other neuromuscular disorders. As infiltration of muscle tissue by activated immune cells is a pathologic mechanism shared between IIM and muscular dystrophies, it is necessary to dig deeper into the differences between immunopathogenic processes and their underlying innate and adaptive immune responses. Seeing cytokines represent key mediators of immune cell function, differentiation and recruitment, they could indeed represent useful disease biomarkers. Determining circulating levels of well-chosen disease-associate cytokines might offer valuable additional information for IIM diagnosis and may orient diagnosis toward or away from a genetic muscle disorder and aid to preselect patients in which causal gene defect should be sought for. Compared to a muscle biopsy, blood sampling is a more convenient diagnostic procedure, it is minimally invasive, technically unchallenging and relatively rapid. Blood samples are routinely available, as serum CK levels represent a well-established diagnostic marker implemented in all medical centers. Ease of sampling also allows periodic monitoring to evaluate disease progression and therapeutic response. A great deal can be expected of further development of circulating cytokines as multi-biomarkers for muscle disorders, yet further validation and larger-scale analyses are necessary. As an important bonus, cytokine profiling of patients will advance our knowledge of immune mechanisms and interactions and help to further elucidate the pathophysiologic pathways underlying the IIM.

## Data availability statement

The original contributions presented in the study are included in the article/supplementary material, further inquiries can be directed to the corresponding author.

## Ethics statement

The studies involving human participants were reviewed and approved by Ghent University Hospital Ethics Committee. The patients/participants provided their written informed consent to participate in this study.

## Author contributions

BDP was responsible for study concept and design, data gathering, analysis, and interpretation, and preparation and approval of the manuscript.

## Conflict of interest

The author declares that the research was conducted in the absence of any commercial or financial relationships that could be construed as a potential conflict of interest.

## Publisher’s note

All claims expressed in this article are solely those of the authors and do not necessarily represent those of their affiliated organizations, or those of the publisher, the editors and the reviewers. Any product that may be evaluated in this article, or claim that may be made by its manufacturer, is not guaranteed or endorsed by the publisher.
